# On combining family- and population-based sequencing data

**DOI:** 10.1186/s12919-016-0026-9

**Published:** 2016-10-18

**Authors:** Yuriko Katsumata, David W. Fardo

**Affiliations:** Department of Biostatistics, University of Kentucky College of Public Health, 111 Washington Ave, Lexington, KY 40536-0003 USA

## Abstract

Several statistical group-based approaches have been proposed to detect effects of variation within a gene for each of the population- and family-based designs. However, unified tests to combine gene-phenotype associations obtained from these 2 study designs are not yet well established. In this study, we investigated the efficient combination of population-based and family-based sequencing data to evaluate best practices using the Genetic Analysis Workshop 19 (GAW19) data set. Because one design employed whole genome sequencing and the other whole exome sequencing, we examined variants overlapping both data sets. We used the family-based sequence kernel association test (famSKAT) to analyze the family- and population-based data sets separately as well as with a combined data set. These were compared against meta-analysis. Using the combined data, we showed that famSKAT has high power to detect associations between diastolic and/or systolic blood pressures and the genes that have causal variants with large effect sizes, such as *MAP4*, *TNN*, and *CGN*. However, when there was a considerable difference in the powers between family- and population-based data, famSKAT with the combined data had lower power than that from the population-based data alone. The famSKAT test statistic for the combined data can be influenced by sample imbalance from the 2 designs. This underscores the importance of foresight in study design as, in this situation, the greatly lower sample size in the family-based data essentially serves to dilute signal. We observed inflated type I errors in our simulation study, largely when using population-based data, which might be a result of principal components failing to completely account for population admixture in this cohort.

## Background

Whole genome and whole exome sequencing studies provide the resolution necessary to identify both common and rare genetic variants associated with complex disease phenotypes. It is well known, however, that single-variant tests are underpowered for rare variants, and several group-based approaches have been proposed to address this [1–3]. In addition to combining association signals across a genetic region/group (eg, a gene), it is often necessary to combine these signals across, sometimes disparate, data sets. This is frequently done via meta-analysis where summary statistics are calculated within each data set and aggregated to conduct inference [4]. An alternative is mega-analysis [5, 6] where raw data are shared between studies. To conduct mega-analysis, the statistical framework for each study must be the same, which poses difficulty when some component studies are family-based and others recruit only unrelated individuals. There is currently no consensus on which approach is superior, and the comparison most likely depends to a large degree on the specific setting and various unknowns such as the study-specific genetic architectures.

A popular test for conducting region-based association testing, the sequence kernel association test (SKAT) [7, 8], was recently extended to handle family-based studies. The family-based SKAT (famSKAT) [9] introduces a random effect for family that incorporates familial relatedness and can be used robustly across study designs. Because it is unknown how to best combine across study designs in this context, we explore here various approaches with the flexible famSKAT test and meta-analysis. Using the Genetic Analysis Workshop 19 (GAW19) simulation data, we investigate the combination of population-based (ie, unrelated subjects only) and family-based studies via famSKAT and meta-analysis to evaluate best practices when both data types are available for a particular phenotype of interest.

## Methods

### Data sets

#### Population-based genotype data

Exome sequencing data from part of the Type 2 Diabetes Genetic Exploration by Next-generation sequencing in Ethnic Samples (T2D-GENES) Project 1 were provided for GAW19. The data set includes variant call format (VCF) files for odd-numbered chromosomes from 1943 Hispanic people consisting of 490 from the San Antonio Family Heart Study, San Antonio Family Diabetes/Gallbladder Study, Veterans Administration Genetic Epidemiology Study, and the Investigation of Nephropathy and Diabetes Study family component, and 1453 in Starr County, Texas.

#### Family-based genotype data

Whole genome sequencing data were provided by T2D-GENES Project 2: San Antonio Mexican American Family Studies. As with the population-based genotype data, this data set includes VCF files for odd-numbered chromosomes from 464 sequenced individuals comprising 16 distinct pedigrees.

#### Phenotype data and covariates

We evaluated diastolic (DBP) and systolic blood pressure (SBP) from the first examination using the 200 family- and population-based simulation replicates. We performed principal component analysis (PCA) [10] to detect outliers for each data set, and excluded 1 subject from the population-based cohort and 4 subjects from the family-based cohort. After removing subjects with missing data (81 missing age in the population-based cohort and 69 subjects with incomplete data in the family-based cohort), we analyzed 2252 subjects (1861 in the population-based and 391 in the family-based data sets). We then reran the PCA for each analysis type (ie, for the population-based only, the family-based only, and the combined). We used age, gender, hypertension medication use, the interaction between age and gender, and the top 3 principal components (PCs) as covariates throughout.

#### Statistical analysis

Because the variants in each data set varied, we extracted a data set of intersecting variants based on marker position. VCFtools v0.1.12 [11] was used to obtain biallelic single nucleotide variants (SNVs) from each investigated gene. *P* values for the famSKAT tests were calculated by Kuonen’s method [12] with the R package famSKAT (https://www.hsph.harvard.edu/han-chen/2014/07/31/famskat/). We performed famSKAT for analyzing the family- and population-based data sets separately, as well as for the combined data set. We further combined the famSKAT analyses from population- and family-based designs meta-analytically [13] as implemented in R’s seqMeta package [14]. We used the default weights in famSKAT such that $\sqrt{w_j}$ follows $Beta\left({\hat{MAF}}_j,1,25\right)$ with the sample minor allele frequency $\hat{(MAF)}$ estimated using all subjects.

## Results

### Simulated data

We focused on the top 15 causal genes influencing each of DBP and SBP in the family data set. Variants within 50 kb upstream and downstream of each gene were extracted. Table 1 shows the number of variants in each gene used in the analysis for each of the data sets. We used the 200 simulated phenotype replicates to assess empirical type I error rates and powers.

**Table 1:** The number of variants in each gene in family-based, population-based, and combined data sets

Gene	Number of variants	Number of causal variants		
	Total^a^	Family^b^	Population^c^	Combined^d^
DBP				
*MAP4*	41	5	8	8
*TNN*	52	11	15	15
*NRF1*	17	0	0	0
*LEPR*	43	3	7	7
*FLT3*	46	1	2	2
*ZFP37*	18	1	5	5
*CGN*	56	9	16	16
*MTRR*	62	6	10	10
*SLC35E2*	29	0	0	0
*ZNF443*	20	1	5	5
*RAI1*	55	3	7	7
*PTTG1IP*	47	0	0	0
*CABP2*	37	1	0	1
*ZNF544*	34	3	3	3
*REPIN1*	32	3	4	4
SBP				
*MAP4*	41	5	9	9
*TNN*	52	12	15	15
*NRF1*	17	0	0	0
*LEPR*	43	3	7	7
*FLT3*	46	1	2	2
*ZNF443*	20	1	5	5
*CABP2*	37	1	0	1
*GTF2IRD1*	34	0	0	0
*FLNB*	81	5	7	7
*GSN*	39	2	7	7
*LRP8*	35	1	2	2
*PSMD5*	33	2	4	4
*GAB2*	77	1	2	2
*ABTB1*	42	1	1	1
*KRTAD11-1*	4	0	0	0

^a^The number of variants that have the same position between the family- and population-based data

^b^The number of causal variants of the intersected variants in the family-based data

^c^The number of causal variants of the intersected variants in the population-based data

^d^The number of causal variants of the intersected variants in the combined data between the family- and population-based data

### Type I error simulation

To investigate type I error (false-positive) rates, we used variable Q1, which is a heritable quantitative trait without any direct association with genotype. Table 2 shows the empirical type I error rates from the family-based, population-based, and combined data sets, as well as that from aggregating family and population results via meta-analysis. For the family- and population-based designs, the empirical type I error rates were acceptable, ranging from 0.025 to 0.090 and 0.030 to 0.100, respectively. The famSKAT for the combined data and the meta-analytic approach exhibited more inflated type I error rates: 0.050 to 0.135 and 0.055 to 0.130, respectively.

**Table 2:** Type I errors (95 % confidence intervals) of family-based sequence kernel association test in the family-based, population-based, and the combined data, and of meta-analytic approach

Gene	Family	Population	Combined	Meta-analysis
*MAP4*	0.050 (0.024–0.090)	0.100 (0.062–0.150)	0.085 (0.050–0.133)	0.095 (0.058–0.144)
*TNN*	0.030 (0.011–0.064)	0.080 (0.046–0.127)	0.085 (0.050–0.133)	0.125 (0.083–0.179)
*NRF1*	0.050 (0.024–0.090)	0.070 (0.039–0.115)	0.080 (0.046–0.127)	0.065 (0.035–0.109)
*LEPR*	0.040 (0.017–0.077)	0.045 (0.021–0.084)	0.080 (0.046–0.127)	0.055 (0.028–0.096)
*FLT3*	0.090 (0.054–0.139)	0.060 (0.031–0.102)	0.060 (0.031–0.102)	0.070 (0.039–0.115)
*ZFP37*	0.045 (0.021–0.084)	0.045 (0.021–0.084)	0.060 (0.031–0.102)	0.110 (0.070–0.162)
*CGN*	0.040 (0.017–0.077)	0.060 (0.031–0.102)	0.070 (0.039–0.115)	0.105 (0.066–0.156)
*MTRR*	0.035 (0.014–0.071)	0.030 (0.011–0.064)	0.065 (0.035–0.109)	0.075 (0.043–0.121)
*SLC35E2*	0.085 (0.050–0.133)	0.065 (0.035–0.109)	0.090 (0.054–0.139)	0.100 (0.062–0.150)
*ZNF443*	0.075 (0.043–0.121)	0.060 (0.031–0.102)	0.050 (0.024–0.090)	0.070 (0.039–0.115)
*RAI1*	0.035 (0.014–0.071)	0.090 (0.054–0.139)	0.080 (0.046–0.127)	0.095 (0.058–0.144)
*PTTG1IP*	0.070 (0.039–0.115)	0.080 (0.046–0.127)	0.095 (0.058–0.144)	0.110 (0.070–0.162)
*CABP2*	0.055 (0.028–0.096)	0.090 (0.054–0.139)	0.090 (0.054–0.139)	0.130 (0.087–0.185)
*ZNF544*	0.045 (0.021–0.084)	0.080 (0.046–0.127)	0.090 (0.054–0.139)	0.095 (0.058–0.144)
*REPIN1*	0.065 (0.035–0.109)	0.050 (0.024–0.090)	0.075 (0.043–0.121)	0.080 (0.046–0.127)
*GTF2IRD1*	0.080 (0.046–0.127)	0.075 (0.043–0.121)	0.060 (0.031–0.102)	0.080 (0.046–0.127)
*FLNB*	0.050 (0.024–0.090)	0.070 (0.039–0.115)	0.095 (0.058–0.144)	0.120 (0.078–0.173)
*GSN*	0.065 (0.035–0.109)	0.055 (0.028–0.096)	0.055 (0.028–0.096)	0.105 (0.066–0.156)
*LRP8*	0.065 (0.035–0.109)	0.050 (0.024–0.090)	0.135 (0.091–0.190)	0.105 (0.066–0.156)
*PSMD5*	0.025 (0.008–0.057)	0.080 (0.046–0.127)	0.080 (0.046–0.127)	0.090 (0.054–0.139)
*GAB2*	0.030 (0.011–0.064)	0.065 (0.035–0.109)	0.065 (0.035–0.109)	0.105 (0.066–0.156)
*ABTB1*	0.075 (0.043–0.121)	0.075 (0.043–0.121)	0.100 (0.062–0.150)	0.125 (0.083–0.179)
*KRTAD11-1*	0.070 (0.039–0.115)	0.055 (0.028–0.096)	0.050 (0.024–0.090)	0.055 (0.028–0.096)

### Power simulation

**Fig. 1:**
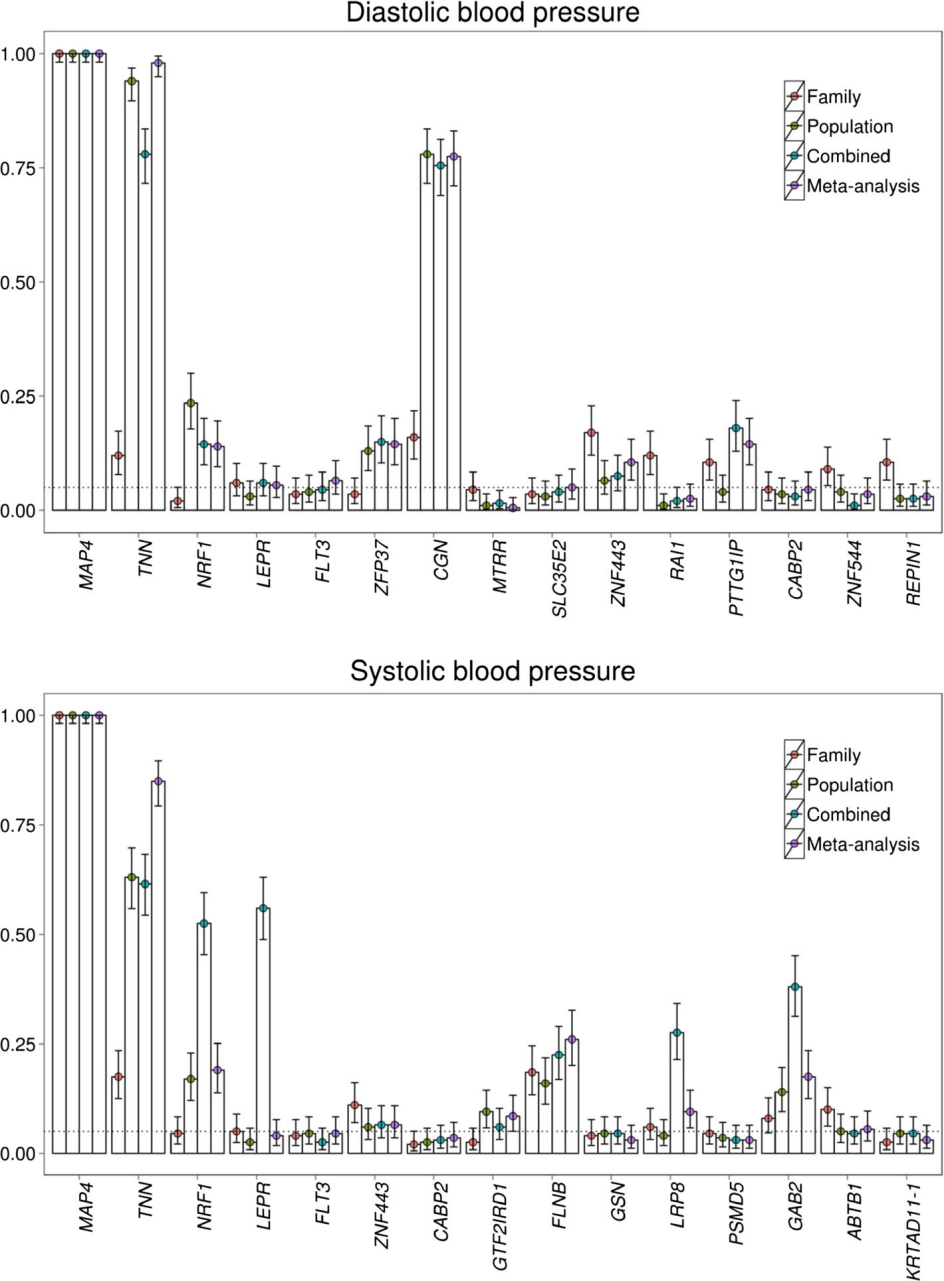
Powers of family-based sequence kernel association test (famSKAT)

Figure 1 shows the simulation results for power for DBP and SBP at α = 0.05 in family- and population-based designs alone, the combined data approach, and the meta-analytic approach. For DBP, the famSKAT had high power to detect the *MAP4*, *TNN,* and *CGN* genes in the combined data set (*MAP4*: 1.00; *TNN*: 0.780; *CGN*: 0.755); however, the tests in the combined data had lower power than those in the population-based data focusing on the *TNN* and *CGN* genes (*TNN*: 0.940; *CGN*: 0.780). For SBP, the famSKAT had high to moderate power to detect the *MAP4*, *TNN*, *NPE1,* and *LEPR* genes (*MAP4*: 1.00; *TNN*: 0.615; *NPF1*: 0.525; *LEPR*: 0.560) and lower power to detect the *FLNB*, *LRP8,* and *GAB2* genes (*FLNB*: 0.225; *LRP8*: 0.275; *GAB2*: 0.380) in the combined data.

## Discussion

In this study, we investigated the combination of population-based and family-based data via famSKAT to evaluate best practices when both data types are available for a particular phenotype of interest. We showed in simulation studies that famSKAT using the combined data had high power to detect association between DBP and/or SBP and the genes that have causal variants with large effect sizes and had similar levels of power with the meta-analytic approach for most genes. Notably, meta-analysis substantially outperforms the combined data approach for only *TNN*, while combining data is substantially better for *NRF1*, *LEPR*, *LRP8* and *GAB2*. Interestingly for these 4 genes, the power gain is sizable (eg, gain of 52 % power by combining data for *LEPR*). However, when there was a considerable difference in the powers between family- and population-based data, famSKAT in the combined data had lower power than that in the population-based data alone. For example, the *TNN* gene for both DBP and SBP and the *CGN* gene for DBP in the combined data had lower power than in the population-based data. The power of famSKAT in the combined data is more affected by extremely low power in either data set (family-based in this case) compared to the meta-analytic approach. The application of famSKAT to the GAW19 data demonstrates that combining family- and population-based data did not improve the power to detect the *TNN, CGN* genes compared with the power from the population-based design only.

The famSKAT test statistic for the combined data can be influenced by sample imbalance from the 2 designs. This underscores the importance of foresight in study design as, in this situation, the greatly lower sample size in the family-based data essentially serves to dilute signal. As a result of difficulty in subject recruitment and high costs of sequencing, family-based studies tend to have smaller sample. In addition, our simulation study shows that both approaches to combine studies, famSKAT with combined data and meta-analysis, had inflated type I error. These inflated type I errors, which were largely when using population-based data, might be a result of unaccounted for population admixture, even when adjusting for PCs.

## Conclusions

The famSKAT test, when combining population-based and family-based data, had high power to detect an association between DBP and/or SBP and the genes that have causal variants with large effect sizes. It had similar levels of power with the meta-analytic approach for most genes. However, the power of famSKAT in the combined data was more affected by extremely low power in either data set compared to the meta-analytic approach. The famSKAT test statistic for the combined data can be influenced by sample imbalance from the two designs. This underscores the importance of foresight in study design as, in this situation, the greatly lower sample size in the family-based data essentially serves to dilute signal.
